# Natal soil consumption shifts gut microbiome in captive Ōkārito kiwi (*Apteryx rowi*)

**DOI:** 10.1186/s42523-025-00445-5

**Published:** 2025-08-06

**Authors:** Stephen P. Rowe, Matthew B. Stott, Bethany Brett, Priscilla A. San Juan, Anastasija Podolyan, Manpreet K. Dhami

**Affiliations:** 1 Te Kura Pūtaiao Koiora| School of Biological Sciences, Te Whare Wānanga o Waitaha| University of Canterbury, Ōtautahi| Christchurch, Aotearoa, New Zealand; 2 Biocontrol & Molecular Ecology, Manaaki Whenua Landcare Research, Lincoln, New Zealand; 3 Willowbank Wildlife Reserve, Christchurch, New Zealand; 4 Department of Mammalogy, Research and Collections, Natural History Museum of Los Angeles County, Los Angeles, USA; 5 School of Biological Sciences, Waipapa Taumata Rau, University of Auckland, Auckland, New Zealand

**Keywords:** Probiotics, Conservation, Avian gut Microbiome

## Abstract

**Background:**

Captive-rearing programmes for endangered birds, such as those in place for kiwi conservation in Aotearoa-New Zealand, can unintentionally deprive the birds access to a microbially-diverse and ‘natural’ developmental environment i.e., their natal *rohe* (territory). These programmes introduce external variables such as increased exposure to diseases, unnatural and incomplete diets, antimicrobial usage, and artificial cohabited environments, which have the potential to impact rearing success outcomes. In this research, we investigated whether the introduction of natal soils, as a direct probiotic and a source of wild microorganisms, to the captive-reared ground-foraging Ōkārito kiwi (*Apteryx rowi*) chick diet would impact their gut microbiome. Using 16S rRNA gene and ITS sequencing to identify the key taxonomic groups present, we assess the community composition differences with the introduction of natal soils into the diet of captive-reared Ōkārito kiwi.

**Results:**

Results showed a distinct gut microbial community associated with Ōkārito kiwi in captivity. Bacterial diversity in Ōkārito kiwi gut increased with age, with the relative abundances of dominant taxonomic groups changing over time. Bacterial phyla *Firmicutes*, *Proteobacteria* and *Actinobacteria*, and the fungal orders *Malasseziales* and *Trichosporon* dominated the gut community. Exposure to natal Ōkārito soils influenced the composition of the gut microbiome in Ōkārito kiwi, especially on the temporal trends of key bacterial taxa. Kiwi with natal-soil-amended diets harboured an increased proportion of *Firmicutes* and *Malasseziales* compared to the ‘Control’ group. The fungal community in the Ōkārito kiwi gut was more transitory, changing rapidly following soil amendment. No significant changes to growth rates or overall health were found between ‘Control’ and ‘Treatment’ groups.

**Conclusions:**

The findings of this study represent the first description of the gut microbiome of the critically endangered Ōkārito kiwi, *Apteryx rowi*, and the first documented use of natal soil as a probiotic amendment for wild birds. Results show that mediation of the gut microbial communities of captive-reared ground-foraging birds can be achieved through the introduction of natal soils in their diet.

**Supplementary Information:**

The online version contains supplementary material available at 10.1186/s42523-025-00445-5.

## Introduction

Aotearoa-New Zealand is home to a unique collection of charismatic indigenous fauna such as the kiwi, kākāpō, giant wētā and tuatara. These endemic fauna have evolved without the pressure of terrestrial predators that occur elsewhere due to the island chain’s relative isolation from other large landmasses [1]. Unfortunately, this has resulted in many of NZ’s endemic species being threatened by invasive predators such as rats, stoats, possums, cats, and dogs [1]. In particular, kiwi and kākāpō have been brought to near-extinction, as their flightlessness and ground-nesting habit leaves them vulnerable to predation.

Aotearoa-New Zealand has established conservation programmes to protect endemic species from these threats; predator-free sanctuaries, nationwide trapping and baiting efforts, and captive-rearing of critically endangered species [2, 3]. One such programme, Operation Nest Egg (ONE), has been responsible for the resurgence of wild kiwi in the last 20 years and the removal of the North Island Brown Kiwi (*Apteryx mantelli*) from the IUCN Red List of Threatened Species [4, 5]. The ONE program lifts wild kiwi eggs from their natural habitat and hatches them in wildlife centres across the country, and rears kiwi chicks to adulthood in a predator-free environment before their eventual release [6]. Combined efforts from predator control and ONE programs have been invaluable to species recovery - however, the impacts of captivity on these birds eventually released into the wild remain understudied [7, 8].

Building on the long-term success of the ONE programme, we now have an opportunity to critically consider features of the captive environment that may inadvertently influence the health of captive-reared animals [9]. One area of focus is improving the captive rearing environment where animals, such as kiwi and its lesser studied congener, Ōkārito kiwi (*Apteryx rowi*), are likely to be exposed to unfamiliar microorganisms, are typically fed modified diets, treated with antibiotics, and often co-housed in small, restricted built-environments [10, 11]. These factors are likely to substantially impact the kiwi gut microbiome in a similar manner to that of captive house mice [12], or in the case of incubated and hand-reared sage grouse chicks, result in gut microbiomes significantly dissimilar to adult birds [13]. Similar divergence in gut microbiota in other captive-reared chicks have been observed, with indications that rearing style may alter chick health [14–16].

Gut dysbiosis is commonly defined as a change to the composition of the resident microbial communities compared to those in healthy individuals [17]. This change, often observed as a loss of microbial diversity and ‘beneficial’ microorganisms, and/or expansion of pathogen abundance [17, 18], can increase the chance of illness and may reduce fitness of the animal hosts [17]. Gut dysbiosis in early development is emerging as a contributor in the reduced capacity of the adaptive immune system in birds, such as in Passerines and chickens [11, 19]. In captive Northern Brown Kiwi, gut dysbiosis presents as the loss of bacterial and fungal diversity, and a shift in species composition compared to wild birds, in combination with the lack of the “natal” microbiome [8]. Birds such as Ōkārito kiwi, are particularly vulnerable and in captive conditions known to acquire an array of health issues, including Aspergillosis [20], cloacitis, conjunctivitis, and gastrointestinal issues [21].

A potential solution to alleviating dysbiosis caused by factors such as captivity and diet is the application of probiotics; the intentional introduction of beneficial microorganisms to a host’s biome [22, 23]. Whether implemented as a food additive in domestic poultry [24] or as an environmental reservoir of wild microorganisms in captive frog and salamander habitats [25, 26], probiotics have been shown to improve health outcomes in a variety of animals through attenuation of gut and skin microbial communities. In their natural habitat, kiwi chicks, as young as 3–5 days old, spend the night foraging amongst soil and leaf litter for insects and worms [27], and in the process, inoculate themselves with soil-borne microorganisms. Conversely, in captivity, this foraging behaviour is confined to smaller areas provisioned with farm- or urban-sourced soils, and generally only during the later stages of captive-rearing [7]. Indeed, in the early stages of captive-rearing, during the critical period of microbial acquistion, kiwi chicks are housed in nearly sterile or soil-free environments [28]. Further, the captive diet, while nutritionally balanced [9, 10], likely offers a markedly different microbial profile, composed mainly of beef, cat/dog biscuits, and vitamins instead of a wild diet comprising typically of insects, soil, and foliage [27]. This nutritionally optimised diet also does not encourage natural foraging behaviours of digging and prodding in soil and litter. In this study, we sought to examine the impact of natal soils - soil collected near wild kiwi parental dens - as a probiotic food additive for kiwi chicks in captivity. We hypothesized that the addition of these soils would augment the captive diet with natal soil microorganisms. We note here that the provision of natal soils to the kiwi chick has potential complementary outcomes within *mātauranga Māori* (the Māori knowledge framework), by providing the kiwi chicks with a direct link to *whenua* and *whakapapa* (land and genealogy) which are considered key for *hauora* (health and well-being) within māoridom.

## Methods

**Fig. 1:**
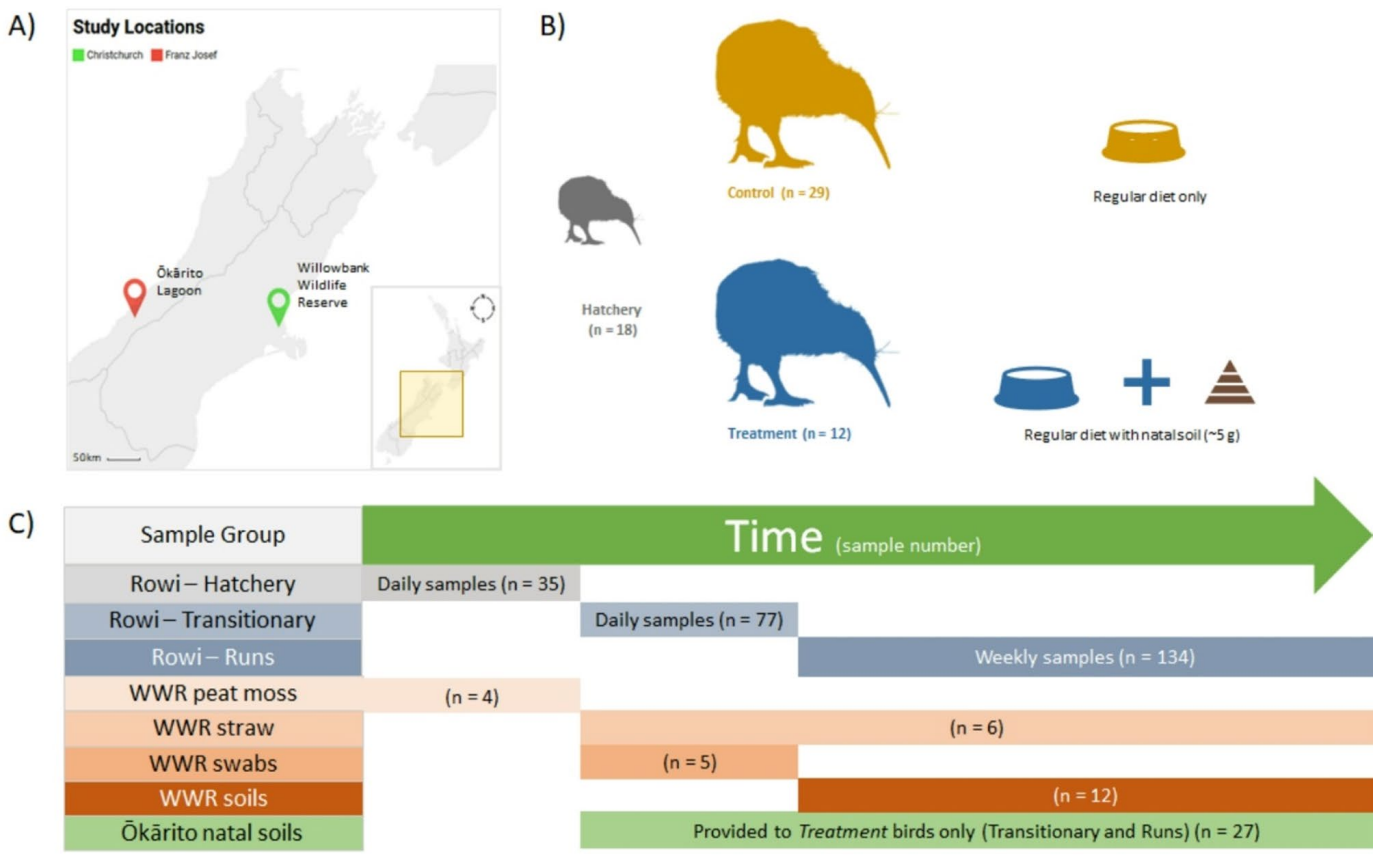
**(A)** A map depicting relevant study locations in New Zealand - The Ōkārito Lagoon on the West Coast and Willowbank Wildlife Reserve (WWR) in Christchurch. **(B)** Feeding regimen of Treatment (orange) and Control (blue) birds. **(C)** A timeline of sampling stages and numbers

Over the 2020 / 2021 summer breeding season, eggs of Ōkārito kiwi (*Apteryx rowi*, also known as rowi) were lifted from their home range in the Ōkārito Reserve on the West Coast (Fig. 1a) and transported to a hatchery in Franz Josef (West Coast Wildlife Center, WCWC) and captive-rearing facility (Willowbank Wildlife Reserve, WWR), as part of the ONE programme. During the captive-rearing period at WWR, Ōkārito kiwi chicks can be highly vulnerable to disease and their nutritional needs are met as specified in the kiwi husbandry protocol, with only small changes permitted. We supplemented natal soils collected from the Ōkārito Reserve into the feeding programme of Ōkārito kiwi chicks and studied its impact on Ōkārito kiwi gut microbiome. Specifically, we determined the bacterial and fungal community composition of Ōkārito kiwi from control and treatment cohorts as well as environmental sources including natal soils and features of the captive environment including bedding material, housing, and established WWR enclosure soils via 16S rRNA gene and ITS amplicon sequencing. A temporal component for the gut microbiome was used to examine changes over time and to establish baseline data of the gut microbiome composition of *A. rowi*. The provision of natal soil also reinforces a cultural link for the translocated Ōkārito kiwi, an approach supported by the Māori *kaitiaki* (guardians) of the Ōkārito kiwi, Te Rūnaka o Makaawhio. Due to the high vulnerability of Ōkārito kiwi chicks in captivity, our manipulations were limited to minor adjustments.

### Experimental cohorts

Ōkārito kiwi hatched or transported as hatchlings from the WCWC to WWR between October 2020 to April 2021 were separated into ‘Treatment’ (*n* = 12) and ‘Control*’* (n = 29) cohorts. Fresh faecal samples (n_Treatment_ = 84, n_Control_= 110) were collected from both cohorts through three captive rearing stages (‘Hatchery’; 0–21 days, ‘Transitionary’; 21–25 days, and ‘Runs’; 25 + days) for microbial community composition determination (Fig. 1). Environmental samples representing the captive environment at WWR, such as Runs soil (n = 12), bedding straw (n = 12), peat moss (n = 6), hutch swabs (n = 5), as well as natal Ōkārito soil (n = 27) were also collected to identify microbial community composition (Fig. 1).

We highlight here that as the Ōkārito kiwi are critically endangered, our ability to control some experimental variables was restricted by management guidelines set by WWR and the New Zealand’s Department of Conservation. In our study, many of the birds were co-housed, which reduced our effective sample size. In addition, when Ōkārito kiwi became sick (as is common with captive co-housing), the chicks were removed from the study to ensure that results were not skewed. In addition, as newly hatched chicks, some birds are exposed to antimicrobials and vaccinations against parasites as preventative care. When this occurred, we accounted for these in our study design by either removing birds with antibiotic exposure, or restricting sampling to periods following recovery period of 2 weeks.

### Natal soil introduction and sample collection

Natal soil introduction began when chicks were in Hatchery. Treatment birds were provisioned with a 7–10 g portion of natal Ōkārito soil added to their standard diet, while Control birds had no changes made to their diet. We note here that prior to exposing the Ōkārito kiwi chicks to the Ōkārito soils, we developed a novel qPCR assay to screen the soil supplements for *Aspergillus fumigatus*, a well-described captive bird pathogen, and found no evidence of the fungus above detectable levels [29]. Faecal samples were collected using either sterile spatulas, swabs, or tweezers depending on material consistency, and placed into sterile 5 mL microcentrifuge tubes (Sigma-Aldrich) with 200-proof molecular-grade ethanol. Soil and substrate samples were collected in the same manner and then stored immediately at -18 °C.

Hatchery and Transitionary stage birds were sampled once daily, while Runs stage birds were sampled weekly. Faecal samples collected from chicks co-housed in pairs were treated as a single bird. Additional sample metadata, including health observations, relevant to each sample is provided in the Supplementary Information S1. DNA was extracted using a NucleoSpin™ 96-Soil Kit (Macherey-Nagel) on the Janus extraction robot (PerkinElmer, Waltham, United States) [8]. Bacterial and fungal amplicon datasets were generated targeting the V4 region of the 16S rRNA gene and the ITS1 regions respectively, using a two-step PCR and dual-indexing approach, as described previously [8]. The multiplexed DNA amplification product concentrations were determined via Qubit dsDNA HS Assay kit (Life Technologies), normalised and purified using Ampure beads (Beckman Coulter) and pooled equivolume, and concentration and library size assessed using a LabChip HT DNA 5 K Assay Kit in conjunction with a LabChip GX Touch HT Nucleic Acid Analyser (Perkin Elmer). The final library was diluted to 4 nM (with 10% PhiX spiked as internal control) and sequenced on the Illumina MiSeq platform using v2 flow cell kit with 250 bp paired-end mode at the Auckland Genomics sequencing facility, at the University of Auckland.

### Bioinformatic workflow

Raw paired-end sequence data were processed on the New Zealand eScience Infrastructure (NeSI) High Performance Computing (HPC) using a combination of published and bespoke scripts, as described previously [8] (Supplementary information S1& S2). Briefly, demultiplexing of raw FASTQ files into their relative sample bins was completed using Claident [30] (v.0.2.2019.05.10). Paired-end reads were merged with PEAR [31] (v.0.9.8) (*p* = 0.001) and filtering of low-quality reads was completed by Claident filtering and QC scripts, applying a Phred quality score cutoff of 30 and a minimum read length of 150 bp. VSEARCH [32] (v.2.21.0) was used to filter noisy reads and cluster clean reads into Operational Taxonomic Units (OTUs) at a sequence similarity threshold of 97%. Chimeras were detected using VSEARCH and removed using both *de novo* and reference-based algorithms [33, 34]. Claident was used to generate a summary table of OTUs × sample metadata, as well as a FASTA file of filtered OTUs which would then be presented to RDP Classifier [34] (v.9) for taxonomic classification. A GenBank-derived 16S rRNA gene sequence training set built into Claident was used to assign taxonomy to OTUs for bacterial reads, and the UNITE fungal ITS set was used for fungal reads [30, 33]. MAFFT [35] (v.7.0) was used to align bacterial and fungal FASTA sequences.

### Statistical analysis

R packages *phyloseq* [36] (v.1.37.0) and *vegan* [37] (v.2.5-7) were used for the majority of the analyses. For bacterial data, outlier samples with more than 30,000 reads were removed to reduce bias. OTUs were filtered at the domain and phylum level to remove potential contaminants such as plastid DNA. Sample read depth and summary statistics were generated using the R base package and represented graphically using *ggplot2* [38] (v.3.3.5). Microbial relative abundances were calculated using raw OTU counts in *phyloseq*, and represented graphically using *ggplot2*, *phylosmith* [39] (v.1.0.6) and *microViz* [40] (v.0.9.0). OTU count data were transformed to relative abundance using *phyloseq.* Beta diversity was calculated between sample groups based on cohort or soil type using the betadisper function in the *vegan* package. A permutational multivariate analysis of variance (PERMANOVA) and Tukey’s Honest Significance Difference test were conducted to determine significant differences in beta diversity between soils as well as cohorts. The data were represented using *ggplot2* and *ggplotly* [41] (v.0.0.1). Nonmetric multidimensional scaling (NMDS) with a Bray-Curtis distance metric was used to calculate distance measures between samples based on OTU abundance, organised into cohorts and soil type. Outliers with high read counts were removed and data were log-transformed using the *microbiome* package (v1.29.0) [42]. In some group analyses, rarefaction via *phyloseq* was used to temper variation further before log-transformation was applied. For enumeration of temporal trends of specific taxa, count data were centered log-ratio normalised in *microbiome* package. OTU counts were plotted against sample counts to find the optimal rarefaction levels. The ordination metrics were calculated using the *phyloseq* and *phylosmith* packages using Bray-Curtis distances. Levene’s test for homogeneity of group variances was performed to test if the assumption for PERMANOVA analysis were met. The adonis2 function in *vegan* (PERMANOVA) was used to test significance levels between groups in the ordinations to determine if the plots gave accurate representations of group dissimilarities. A multinomial species classification method (CLAM) test was performed using *vegan*. OTUs with zero count data for both bacterial and fungal datasets were removed, and the test performed with an alpha cutoff value of 0.005. Control chicks were compared directly to Treatment chicks across all captive rearing stages and the results graphically represented using *ggplot* and *ggplotly*.

Ages and weights of chicks were gathered to calculate growth rates. Where chicks were cohoused, the average weight and age of the birds was used for the sample taken. Raw weights and percentage changes in weight (as a proxy for change in biomass) were both plotted in separate time-series using packages *cowplot* (v.1.1.1) [43] and *ggpubr* (v. 0.4.0) [44]. A linear model was fitted to the data to compare differences in growth rates between cohorts. An Analysis of Variance (ANOVA) was used to check model assumptions and slopes were compared between cohorts using package *lsmeans* [45] (v. 2.30-0).

## Results

A total of 2,164,636 and 598,660 raw paired-end reads were generated for the two amplicons: 16S rRNA gene and ITS, respectively. Following quality control, we retained an average read depth of 5,696 reads/sample for 16S rRNA gene amplicon and 1,290 reads/sample for ITS amplicon (See Figure S1 and S2 for read coverage statistics). In this study, we focus on results from the bacterial community analysis and provide further details for the fungal community assessment, for which treatment responses were inconclusive due to data limitations (Figures S3-S6).

### Soil alters gut bacterial community composition, but not diversity

**Fig. 2:**
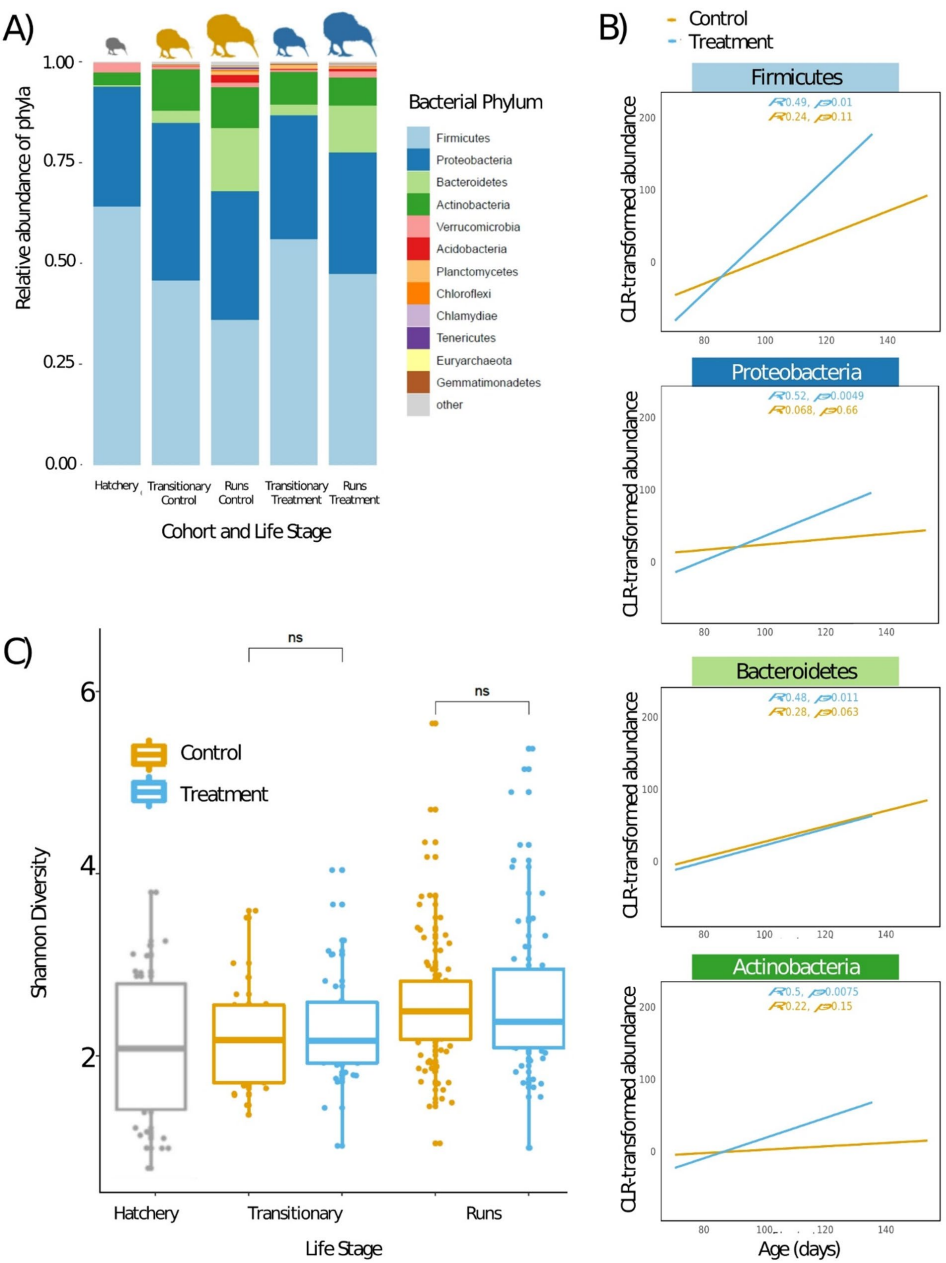
The effect of natal soil on the alpha diversity, community composition and relative abundances of the Ōkārito kiwi gut microbiome over time. (**A**) Relative abundances of the 12 most prominent bacterial phyla over time (Hatchery - Transitionary - Runs) The relative size of the kiwi icons reflects either hatchery chicks (small; grey), transitionary chicks (medium; orange| blue) or chicks in runs (large; orange| blue). (**B**) Immediately following the Transitionary stage, Treatment birds exhibited a sharper increase in the abundance of *Firmicutes* when compared with Control birds. *Proteobacteria* and *Actinobacteria* also increased somewhat differently across Treatment and Control birds, while *Bacteroidetes* shifts were similar. (**C**) Alpha diversity increased as the birds aged (ns; not significant)

Relative bacterial abundances were agglomerated to phylum for each cohort and captive-breeding stage (Fig. 2a; Table 1). The microbiome composition of Hatchery birds were dominated by *Firmicutes* and *Proteobacteria*, which then shifted to include other phyla as the birds aged and were placed into either the Control or Treatment cohort. From there, the Treatment bird microbiomes tended to favour *Firmicutes* which increase sharply (slope *p* = 0.01), whereas Control birds exhibit slower increase in *Firmicutes* (Fig. 2a, b). Shifts in other major taxa such as *Proteobacteria*,* Bacteroidetes*, and *Actinobacteria* were similar across Control and Treatment birds with small but significant shifts observed for *Proteobacteria* and *Actinobacteria* in Treatment birds (Fig. 2b). The bacterial alpha diversity of the gut microbiome increased slightly as the birds aged (Fig. 2c), an observation consistent with their proportional relative abundances (Fig. 2a). No significant difference in alpha diversity between Control and Treatment birds of the same captive-breeding stage were noted (*p* = 0.59). Fungal community composition showed similar trends to those observed in bacteria, with Ōkārito soils more variable between samples but less diverse (Figures S5-S6). However, no significant difference in alpha diversity of fungal taxa were observed between Control and Treatment birds (Figure S4). Fungal community composition was mostly influenced in the Treatment cohort by *Malassezia* and *Trichospora* (Figure S3).

**Table 1:** Changes in relative abundance of each major bacterial phyla per cohort over time. Significance is indicated by asterisks, with *p* < 0.001 = ***

Phylum	Intercept	Cohort	Average age
*Firmicutes*	t = 5.684 *p* = 8.06e-08 ***	t = 1.959 *p* = 0.0522	t = 0.291 *p* = 0.7713
*Proteobacteria*	t = 9.586 *p* = < 2e-16 ***	t = -1.251 *p* = 0.2131	t = -1.826 *p* = 0.0701
*Bacteroidetes*	t = 0.783 *p* = 0.435	t = 0.412 *p* = 0.681	t = 1.978 *p* = 0.050
*Actinobacteria*	t = 4.401 *p* = 2.21e-05 ***	t = -1.491 *p* = 0.138	t = -0.159 *p* = 0.874

### Natal soil bacterial communities contribute to, and shift, the gut bacterial community in ŌkāritoKiwi

**Fig. 3:**
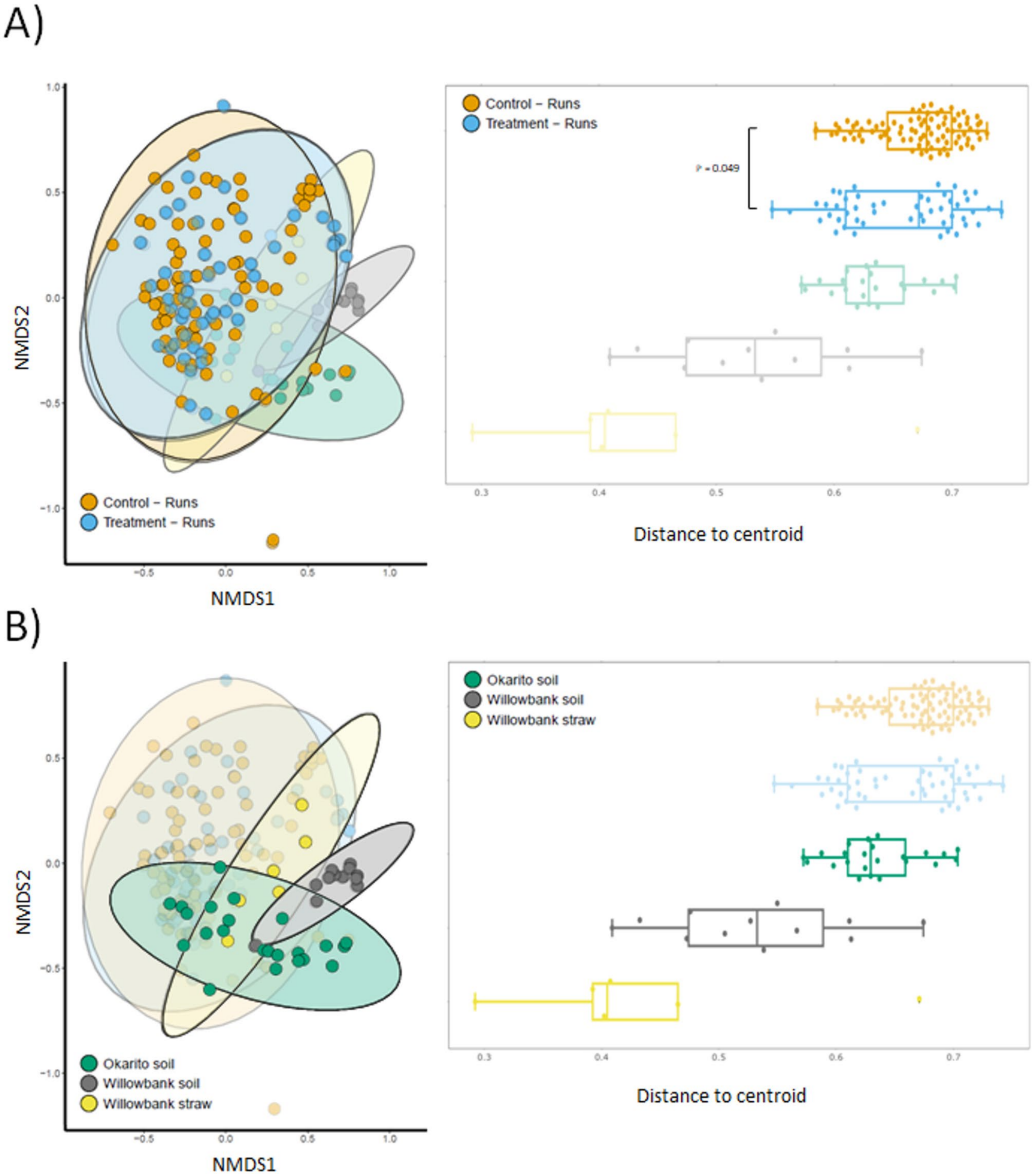
The natal soil microbial community forms the basis for a diverse gut microbiome in captive Ōkārito kiwi populations. For ease of viewing, these data have been separated into (**A**) comparison of Control and Treatment birds, and (**B**) a comparison of soils and bedding material. (**A**) An NMDS and boxplot shows the diversity of the gut bacterial community in Control and Treatment birds at the Runs stage. A larger Distance-to-Centroid indicates higher beta diversity within that group. A significant difference between the Control and Treatment cohort is visible (*p* = 0.049). (**B**) An NMDS and boxplot shows the contribution to Ōkārito kiwi gut microbiome diversity of Ōkārito soil (natal), WWR soil and straw (used as bedding). An overlap can be seen between Ōkārito kiwi and Ōkārito soil, whereas the WWR soil cluster is distinct from the Ōkārito kiwi. Ellipses denote grouping at a 95% confidence level. Fungal community diversity formed clear groups when comparing soils individually but poorly defined groupings when compared with Treatment and Control cohorts (Supplementary Information)

We assessed the natal Ōkārito and captive WWR soils, and the gut microbiome community diversity and composition of birds exposed to the two soil types, to determine whether natal soil consumption impacted the gut microbial community (Fig. 3). Firstly, we found that Ōkārito soils were less diverse (*p* = 0.001) (Figure S7) but more variable from sample to sample (*p* = 7.1e-06) (Fig. 3b) than the WWR soil. In contrast, there appears to be a general overlap between the gut bacterial community composition between Treatment and Control birds (Fig. 3a). Despite this overlap, PERMANOVA analyses showed that the bacterial community composition of Treatment and Control birds was significantly different (*p* = 0.01, *F* = 1.63, df = 1), assumptions for data homoscedasticity satisfied by Levene’s test, *p* = 0.049). Similarly, natal soil bacterial community composition more closely overlapped with the Ōkārito kiwi gut microbiome with a lesser overlap with the Control cohort (Fig. 3b). WWR straw samples, taken from bedding in hutches, appeared to reflect the microbiome of Ōkārito kiwi in general; however, too few samples were collected to draw significant conclusions about their contribution to the Ōkārito kiwi gut microbiome. Small differences in the variance of betadiversity was also observed between Treatment and Control cohorts as the birds aged (Figure S8-S9).

### Natal soil consumption alters the relative abundance of specialist taxa

**Fig. 4:**
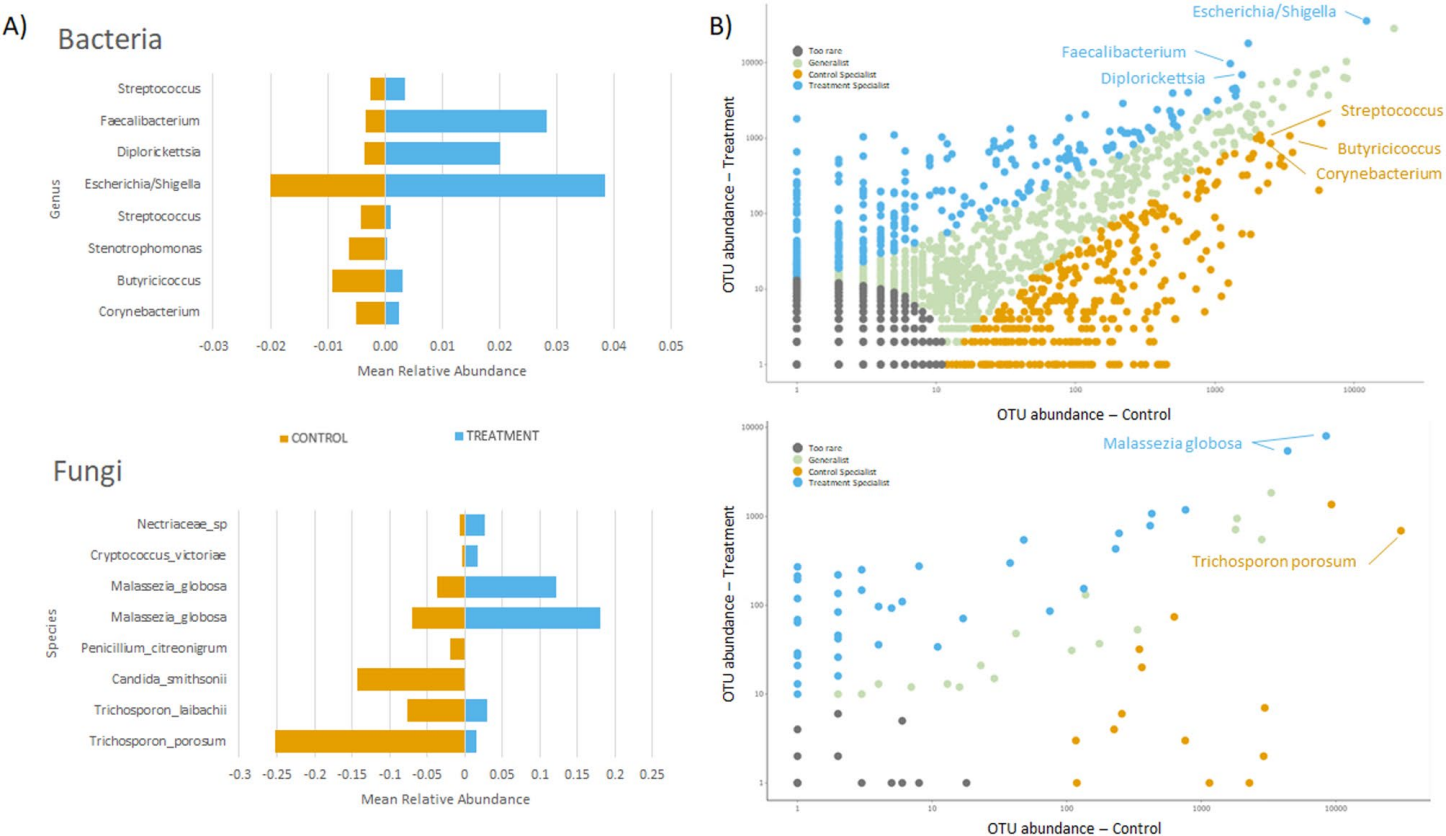
Microbial taxa are differentially abundant in the Ōkārito kiwi gut microbiome following natal soil amendment. (**A**) A SIMPER analysis identified key bacterial and fungal taxa that were responsible for over 70% of the variation between Control and Treatment birds at the Runs stage. The four most prevalent bacterial and fungal taxa in Control and Treatment birds are highlighted. (**B**) CLAMTEST results are plotted on a logarithmic scale for both bacterial and fungal OTUs. Orange points denote ‘Control Specialists’, and blue point ‘Treatment Specialists’

A SIMPER calculation highlighted the observed bacterial and fungal OTUs that were responsible for over 70% of the community composition variation observed between the Control and Treatment cohorts. CLAMtest analysis classified the bacterial and fungal OTUs as predominantly ‘Generalists’, ‘Control Specialists’, ‘Treatment Specialists’, or ‘Too Rare’ (Fig. 4b). The four most abundant bacterial and fungal OTUs identified as either ‘Control Specialists’ or ‘Treatment Specialists’ are highlighted in Fig. 4a along with their average relative abundances in each group. Bacterial OTUs were agglomerated at genus level, and fungal OTUs at species level. The primary ‘Treatment Specialists’ in gut microbiome included bacterial taxa *Faecalibacterium*, *Diplorickettsia* and *Streptococcus*, and the fungal taxa *Malessezia globosa*. Primary ‘Control Specialists’ included representatives of the *Escherichia* / *Shigella*, *Butyricicoccus* and *Corynebacterium* taxa, and the fungi, *Candida smithsonii* and *Trichosporon porosum.*

### Natal soil amendment did not alter kiwi weight gain in the short term

Ōkārito kiwi across the study exhibited similar growth and disease status regardless of their exposure to natal soils. An analysis of covariance of a general linear model used to compare the changes in growth rates over time between Control and Treatment birds found no significant difference in overall average growth rates (7.39 v 7.31 g/day respectively) between the cohorts (*p* = 0.31) (Table S1). Finally, the overall health outcomes such as disease incidence and growth rate were not different between the cohorts (Table S1).

## Discussion

Microbial diversity in the gut is an important factor for the health and wellbeing of animal hosts, including NZ’s endemic kiwi. Once hatched, the nidifugous kiwi chicks immediately begin probing soil and litter in search of insects, which is presumed to act as inocula for their gut microbiome, a feeding behaviour difficult to replicate in captivity [8, 27, 46]. The introduction of natal Ōkārito soils to captive Ōkārito kiwi was hypothesised to help bridge the gap by resembling the microbial dietary inocula in their native habitat. In the same way, the provisioning of natal soils to the newly hatched kiwi chicks also provides an additional cultural link to *whenua* (home lands) that is important within *te ao Māori* (Māori view of the world). We assessed whether access to these natal soils changed the diversity, dominant taxa, or overall composition of bacterial and fungal communities in the gut of captive chicks and found that the amendments to captive diets do alter the gut microbiome of captive chicks, and that natal soils overlap significantly with the Ōkārito kiwi gut microbiome composition. Furthermore, specialist taxa with putative health benefits are differently represented between birds provided with natal soils and those that weren’t.

The relative abundance and alpha diversity measurements show the overall composition of the gut microbiome in Ōkārito kiwi changes over time spent in the captive-breeding programme (Fig. 2a). A definitive trend can be seen as the birds aged - much of this shift is expected as the birds mature and settle into a steady diet [47]. Once chicks begin foraging, the taxonomic complexity of their gut microbiome increases as they intake microorganisms from the environment [24, 26]. In this case, the trend showed a decrease in the overall dominance of two major bacterial phyla, *Proteobacteria* and *Firmicutes*, to make way for less abundant phyla such as *Actinobacteria* and *Bacteroidetes* (Fig. 2a). Importantly, the rate of increase in *Firmicutes* is different across treatment and control birds, with *Firmicutes* increasing in the treatment birds more rapidly once they were introduced to the captive facility soil in the Runs (Fig. 2b). *Firmicutes* have been linked to positive health outcomes in poultry, such as increased growth and immune function [48], as they facilitate the formation of short-chain fatty acids that are directly utilised by the host [49]. Conversely, greater abundance of *Proteobacteria* in Brown kiwi is indicative of the captive-breeding condition [8].

Interestingly, as the two cohorts of birds were exposed to the WWR facility soils (Runs stage), their gut microbiome compositions became more similar. It appears that during the Transitionary stage, the captive chick gut microbiome composition is more prone to variation, particularly for treatment birds (Figure S9), a trend consistent with studies in juvenile captive animals [26, 50], and likely to be affected by the movement of birds from one captive-stage environment to another. The shift in primary bacterial phyla in Treatment chicks could be due to their exposure to natal soils, where *Firmicutes* species that are better suited to niches in the gut microbiome of Okārito kiwi might flourish [51]. Control birds were only exposed to a captive diet and later to a microbially distinct captive facility soil. A recent study found that the microbiome of captive North Island Brown kiwi favoured *Proteobacteria* over *Firmicutes* relative to their wild counterparts; findings that attributed this trend with captivity [8]. The addition of natal soils to the captive diet appears to reverse this trend, shifting community composition towards resembling that of a wild close relative, the Brown Kiwi. Since the Ōkārito kiwi gut microbiome was most similar to the natal soil than to WWR soil, this suggests that soil may be a reservoir for taxa important to the gut microbiome. However, further assessment for wild Ōkārito kiwi samples is required to confirm this.

Microbial composition differences between the two cohorts at the Runs stage showed a similar, but shifted, bacterial community makeup. This difference is attributed to a small supplementation of captive kiwi diet with natal soil, that persists through the duration of the run stage. This finding highlights the potential to shift the gut microbiome community composition via soil amendment. The Ōkārito soils had a greater bacterial beta diversity compared to captive-rearing facility soils, which may be important in recruiting a wider diversity of gut bacteria. Indeed, the minimal overlap of WWR soil with Ōkārito kiwi gut microbiome indicates limited recruitment of gut bacteria from these soils; the importance of an environmental reservoir of functionally relevant microorganisms has been demonstrated in captive studies [25, 26]. While there were no detectable health benefits to natal soil exposure observed in this study, there were no apparent negative health impacts either, supporting the incorporation of natal soil to embed cultural practices as part of ongoing improvements to captive-rearing programmes.

The increase in the relative abundance of *Faecalibacterium* in Treatment cohort was a notable result. This taxon has been associated with better gut health in birds and mammals [8, 51, 52]. However, identifying direct health benefits of microbiome shifts is notably challenging [53], and further longer-term studies are necessary to quantify any persistent health benefits associated with gut microbiome shifts. In contrast to bacteria, we found that gut-associated fungi were transient but were not associated with any particular captive-rearing stage. The common soil associated fungi *Malessezia* spp. increased immediately following exposure to soils [54]. Control chicks exhibited a higher proportion of *Trichosporon* and *Candida* spp. as specialists, both of which are common human-associated fungi [55]. Although both cohorts of chicks were similarly handled by their keepers, the absence of an additional source of microbes (i.e. natal soil) in Control birds emphasize the influence of human dispersed microbiota. However, the low overall read counts of fungal groups in this experiment indicates that further investigation is warranted in order to establish reliable findings.

## Conclusions

The gut microbiome of captive-reared, endangered animals is garnering interest to support long-term conservation outcomes. We confirmed that the Ōkārito kiwi gut microbiome in captivity is shaped by its environment. We further extended this result by incorporating a microbially-rich reservoir of natal soil as a probiotic. We found that this addition of natal soil better reflects the gut bacterial communities of Okārito kiwi and facilitated a shift towards taxa dominance that resembles a wild kiwi gut. Some of these taxa have putative roles in improving bird health, although we did not find evidence of different health outcomes over the course of the study. The supplementation of natal soil into the diet of captive-reared kiwi also has the dual benefit of reinforcing a cultural connection of Ōkārito kiwi to their native environment. Finally, further research is needed to identify the persistence of the shifted gut bacterial community following release into the wild, as well as any long-term health benefits of obtaining potentially beneficial microbes at an early developmental stage.

## Electronic supplementary material

Below is the link to the electronic supplementary material.

Supplementary Material 1

## Data Availability

The dataset(s) supporting the conclusions of this article are available in the DataStore Repository, https://datastore.landcareresearch.co.nz/dataset/natal-soil-kiwi. Te Rūnaka o Makaawhio maintain the cultural authority over these data. Scripts for the bioinformatics processing and statistical analyses are made available at https://github.com/steverowi12/rowi2023.
