# Supplementary material for: Natal soil consumption shifts gut microbiome in captive Ōkārito kiwi (Apteryx rowi)
Source: Anim Microbiome. 2025 Aug 6;7:83. doi: 10.1186/s42523-025-00445-5 (PMC12330010; doi:10.1186/s42523-025-00445-5)
Supplement: Supplementary file 1 — Supplementary Material 1 [file 42523_2025_445_MOESM1_ESM.docx]

### Supplementary Materials for Rowe et al, ‘Natal soil consumption shifts gut microbiome in captive kiwi (*Apteryx rowi*)’

**Supplementary Information**

### **S1** Sample metadata available at <https://github.com/steverowi12/rowi2023.git>

### **S2** Bioinformatic workflow available at <https://github.com/steverowi12/rowi2023.git>

### **S3** RMD file for Bacterial analysis available at https://github.com/steverowi12/rowi2023.git

### **S4** RMD file for Fungal analysis available at https://github.com/steverowi12/rowi2023.git

**Supplementary Figures**


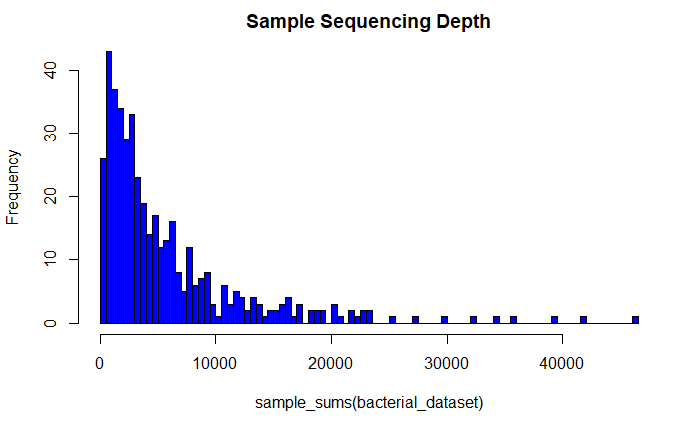


Reads per sample

Frequency


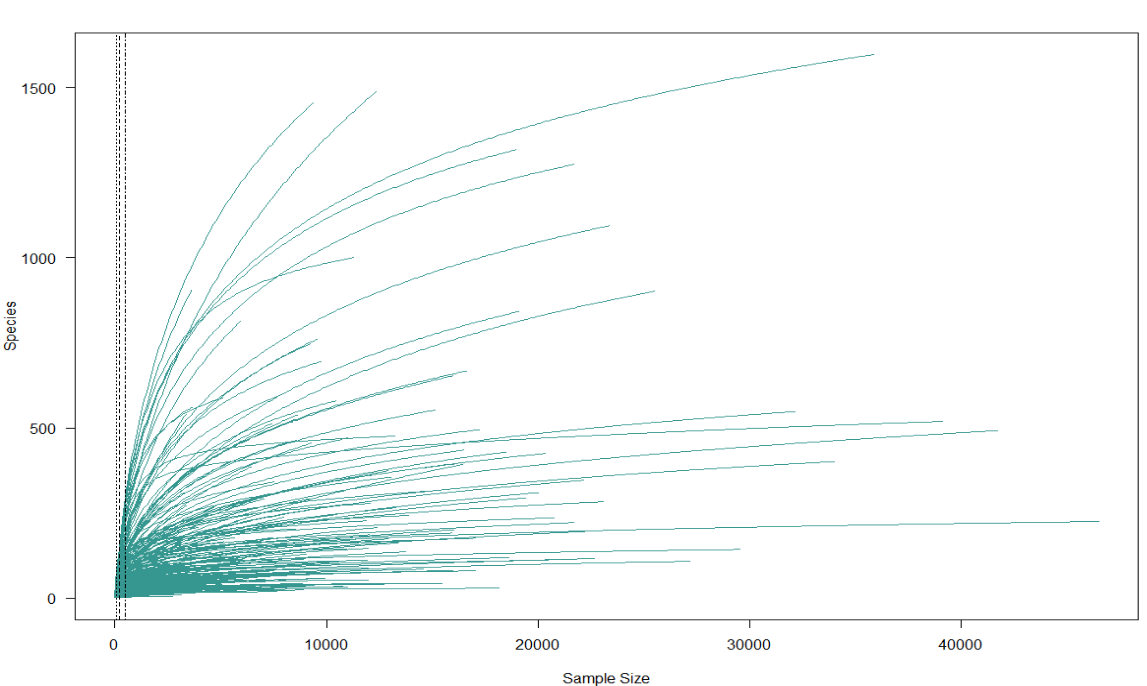


Sample size

OTU

### **Figure S1** Bacterial read depth (top) and rarefaction (bottom)

The average overall sample read depth for all sample types was 5,696 reads per sample, with a maximum read depth of 46,491. For soil samples only, the average read depth was 9,912. Rarefaction was used in some analyses as a transformation method to reduce variation between samples. This was especially important in nonmetric multidimensional scaling (NMDS) calculations to compare different rowi and soil/substrate groups to each other based on differential abundances of taxa in each sample. A rarefaction level of 530 reads per sample was calculated to be the optimum based on retained OTUs and samples post-rarefaction. For the rest of the statistical analyses performed, a simple log10 transformation or Centered-log-ratio (CLR) normalisation of OTU count data was sufficient to reduce bias where needed.


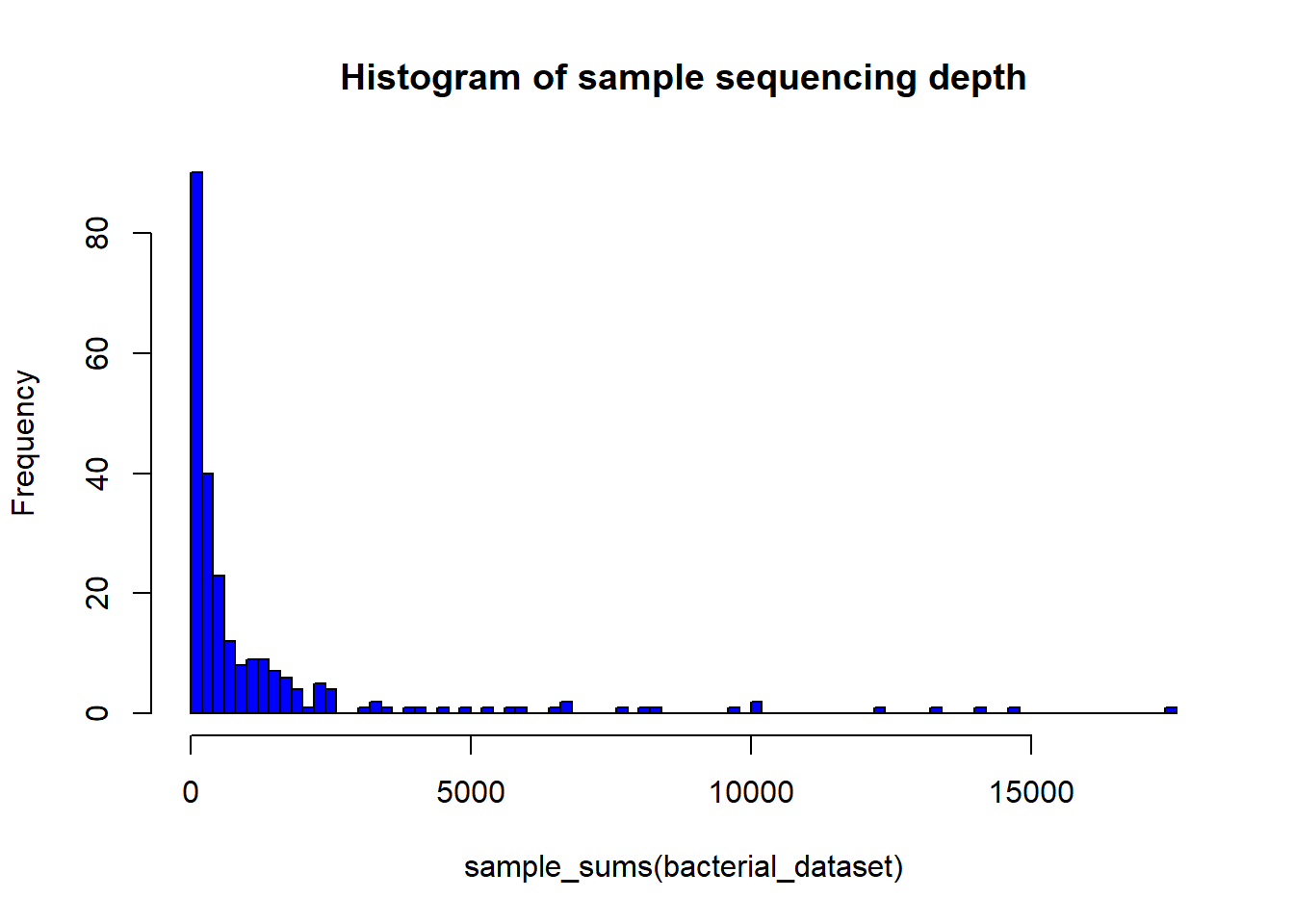


Reads per sample

Frequency


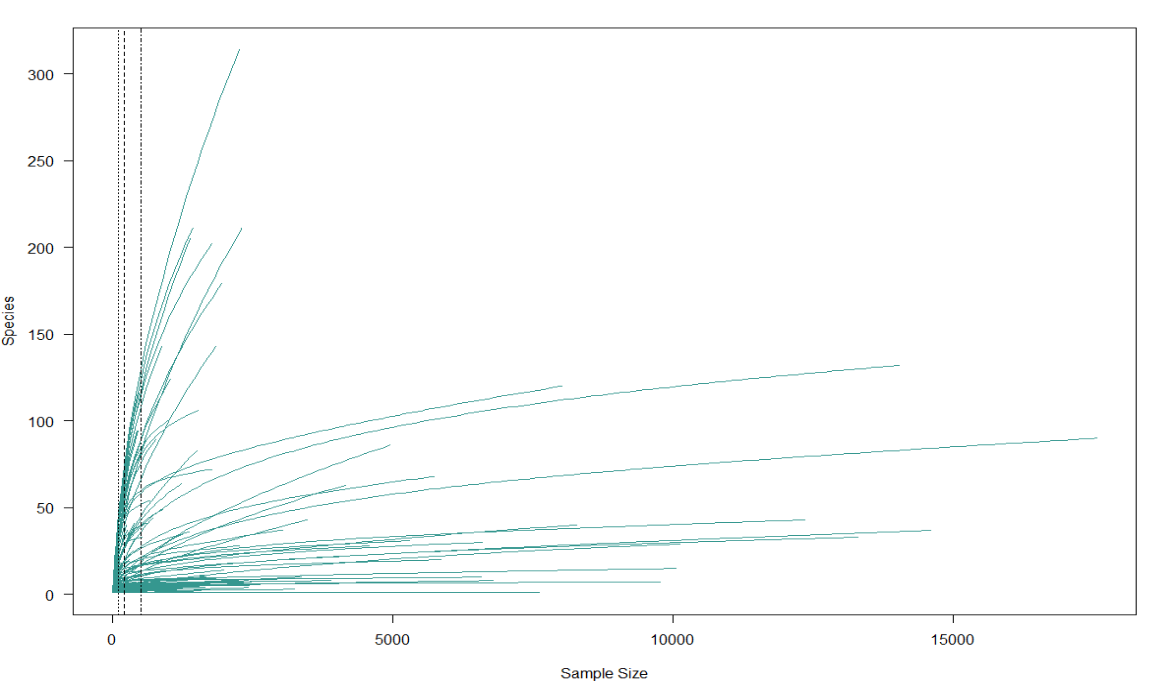


Sample size

OTU

### **Figure S2** Fungal read depth (top) and rarefaction (bottom)

The mean sample read depth for all sample types was 1,290 reads per sample, with a maximum read depth of 17,573 (see Figure 3). For soil samples only, the average read depth was 2,242. As with bacterial data, rarefaction was used as a transformation method in some analyses. A rarefaction level of 100 reads per sample was used (see Figure 4). Log10 transformations were used to minimise variation in analyses that would lose resolution if the data were rarefied. Many of the low read output samples were derived from swabs or fecal samples of very young chicks who were yet to receive first solid meal.


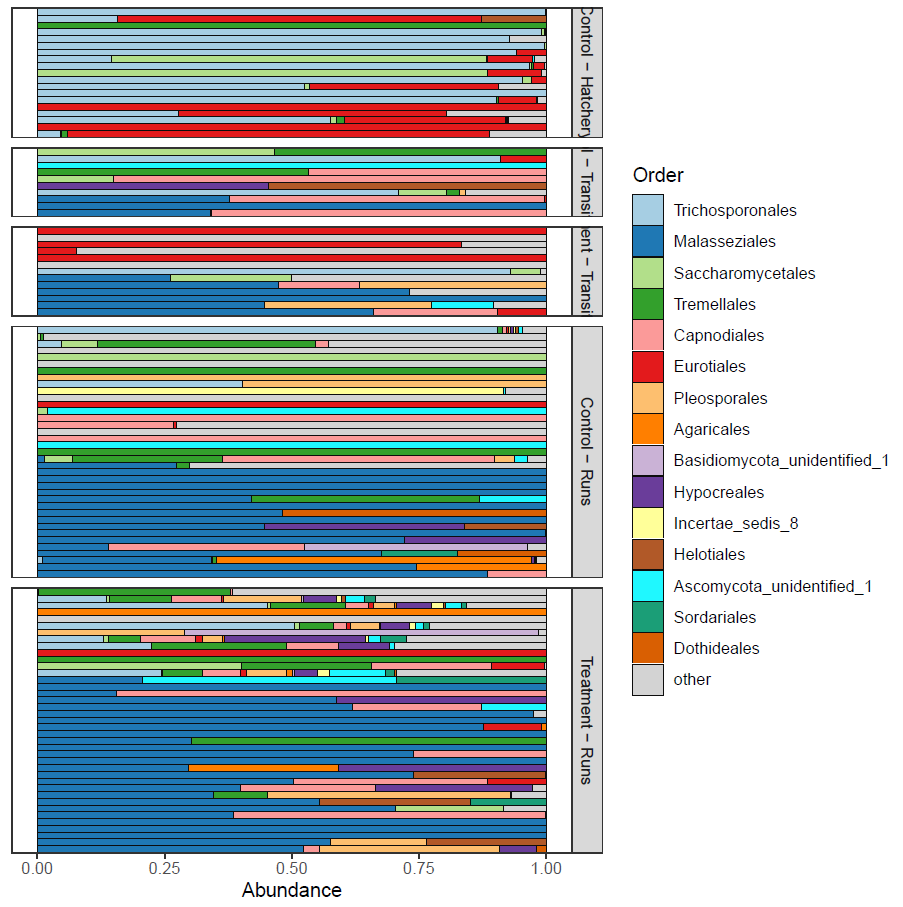


Hatchery

C - T

T - T

**Figure S3** – Relative abundances of fungal orders in individual rowi samples organised by captive-breeding stage and cohort. “C – T” refers to “Control – Transitionary” and “T – T” refers to “Treatment – Transitionary”. A general shift toward a Malasseziales-dominated mycobiome can be seen as the birds age, especially in the Treatment cohort, with a decline in Eurotiales across all cohorts. Control rowi in the Runs stage had a higher prevalence of low-abundance taxa compared to Treatment rowi (other).


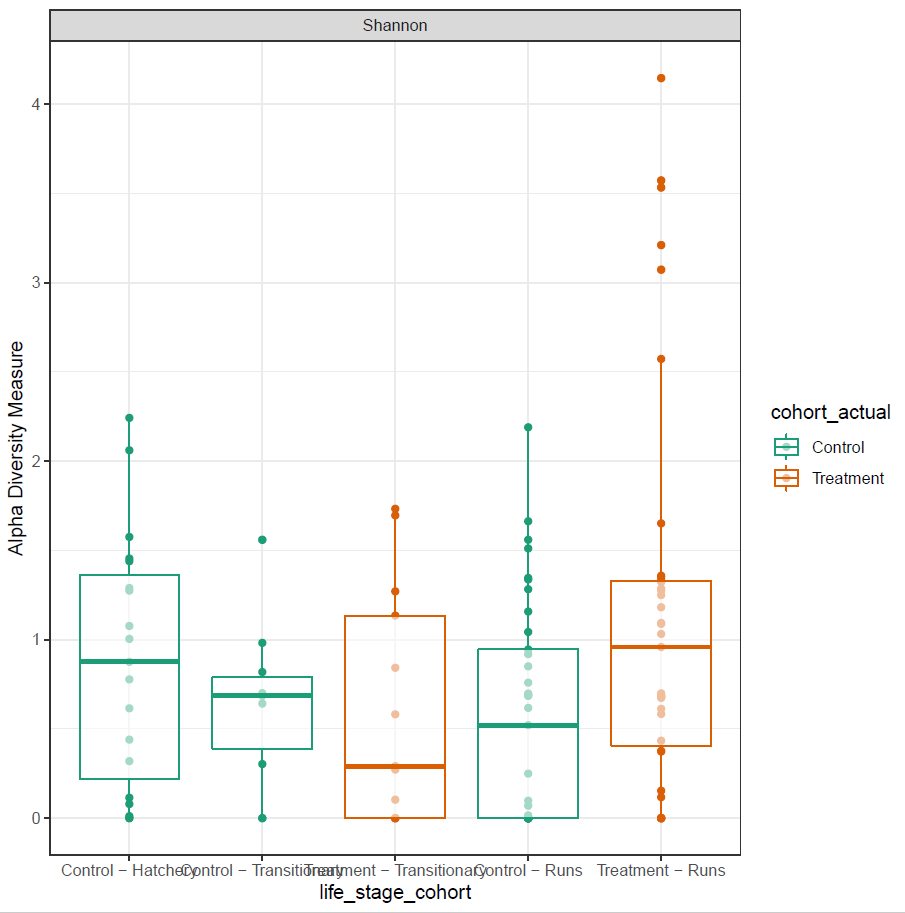

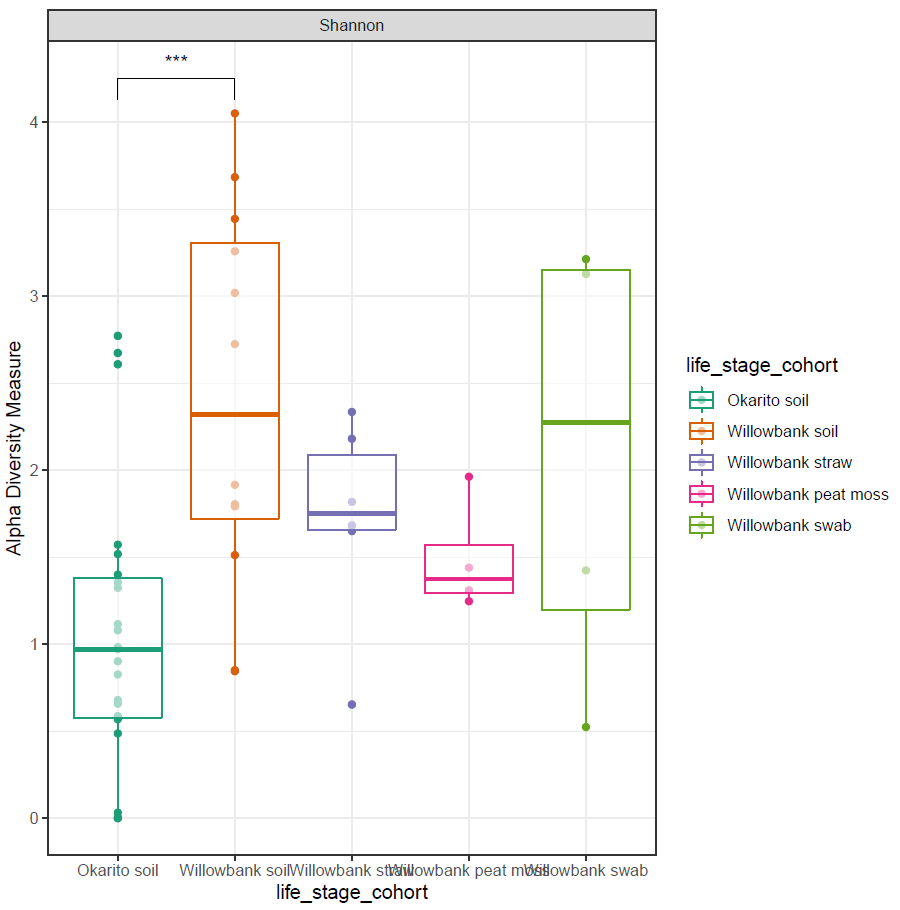


Ōkārito soil

WWR soil

WWR straw

WWR peat moss

WWR swabs

Control Hatchery

Control Transitionary

Treatment Transitionary

Control Runs

Treatment Runs

**B)**

**A)**

**Soil Type**

**Cohort**

Shannon Diversity

Shannon Diversity

**Figure S4** Fungal alpha (Shannon) diversity, colour-coded by soil group, captive-breeding stage and cohort. **A)** A minorly significant difference in alpha diversity within samples was found between Control and Treatment rowi in the Runs stage (p=0.016, TMCM). Overall within-sample fungal diversity increased as the rowi aged. **B)** WWR soil displayed high taxonomic diversity within samples when compared with other soils and substrates. A significant difference in alpha diversity was found between Ōkārito soil and WWR soil as denoted by *** (p=0.008, TMCM).


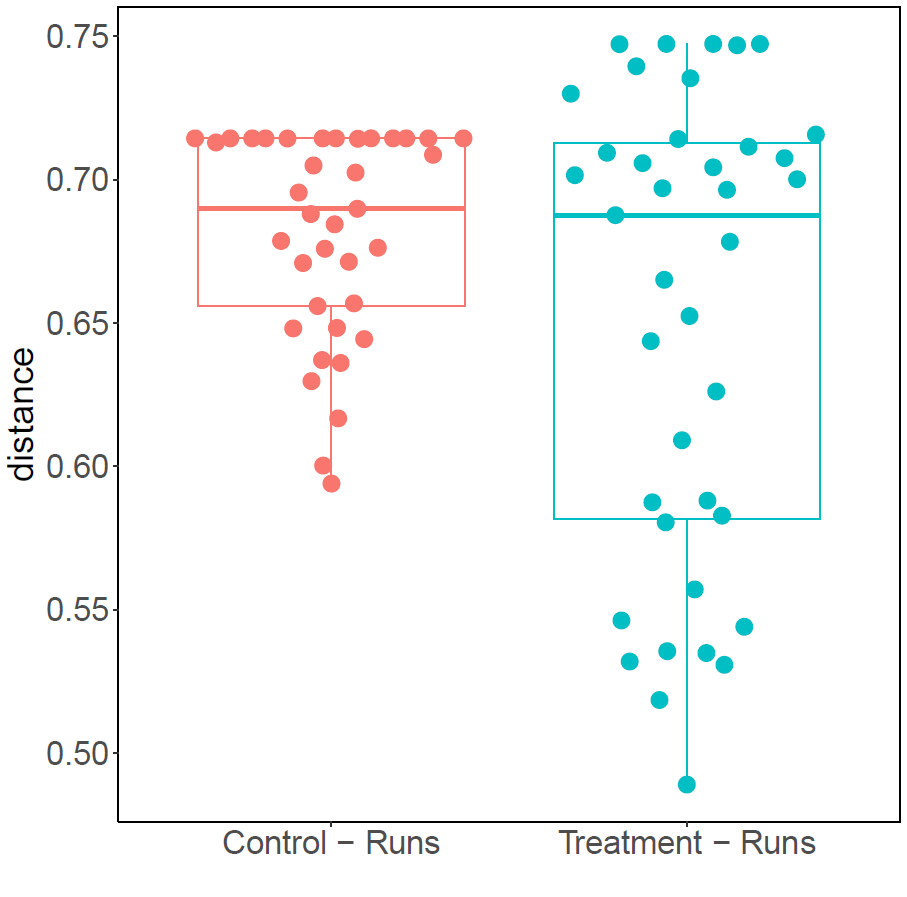

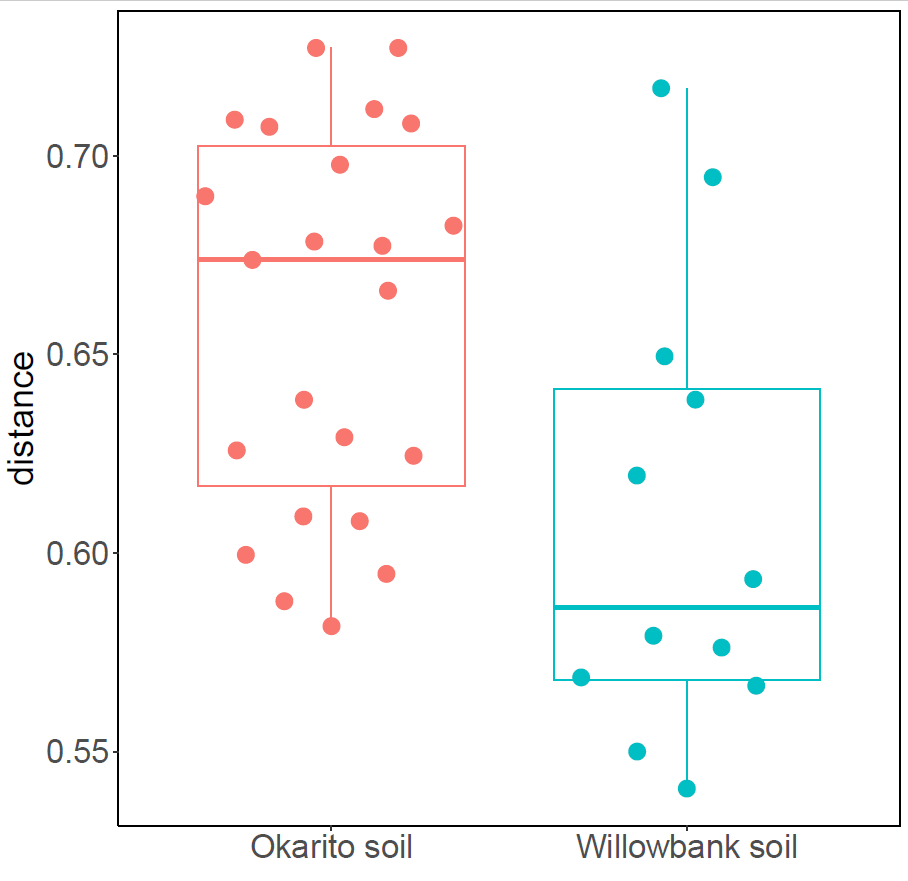

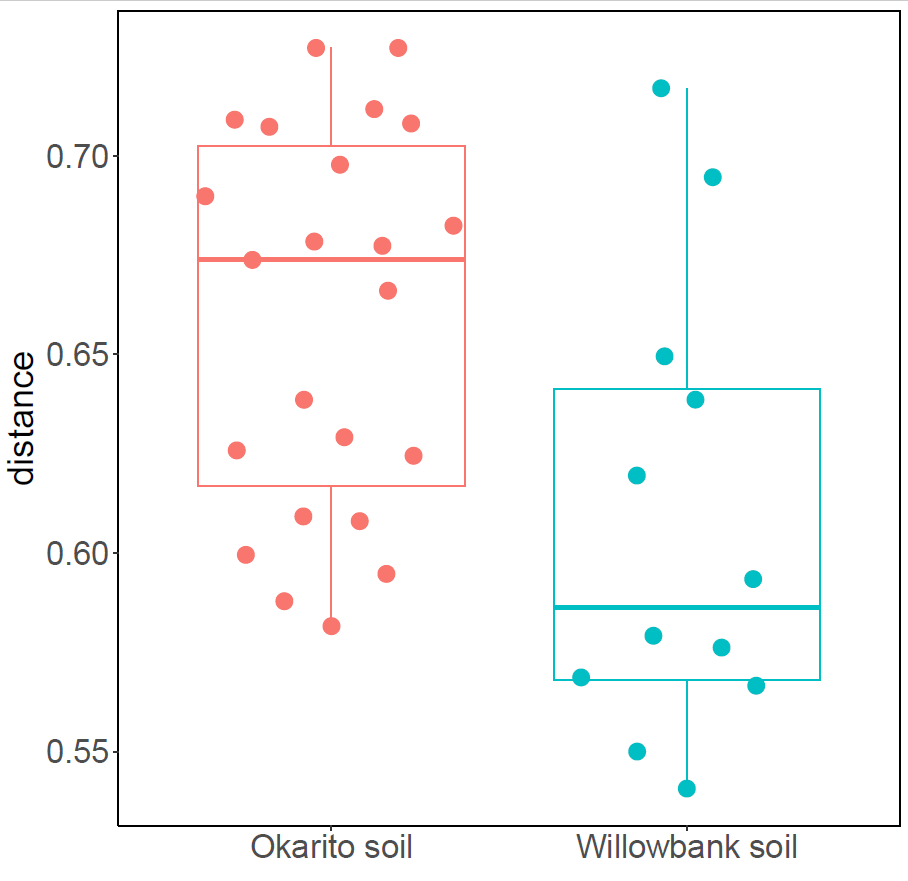

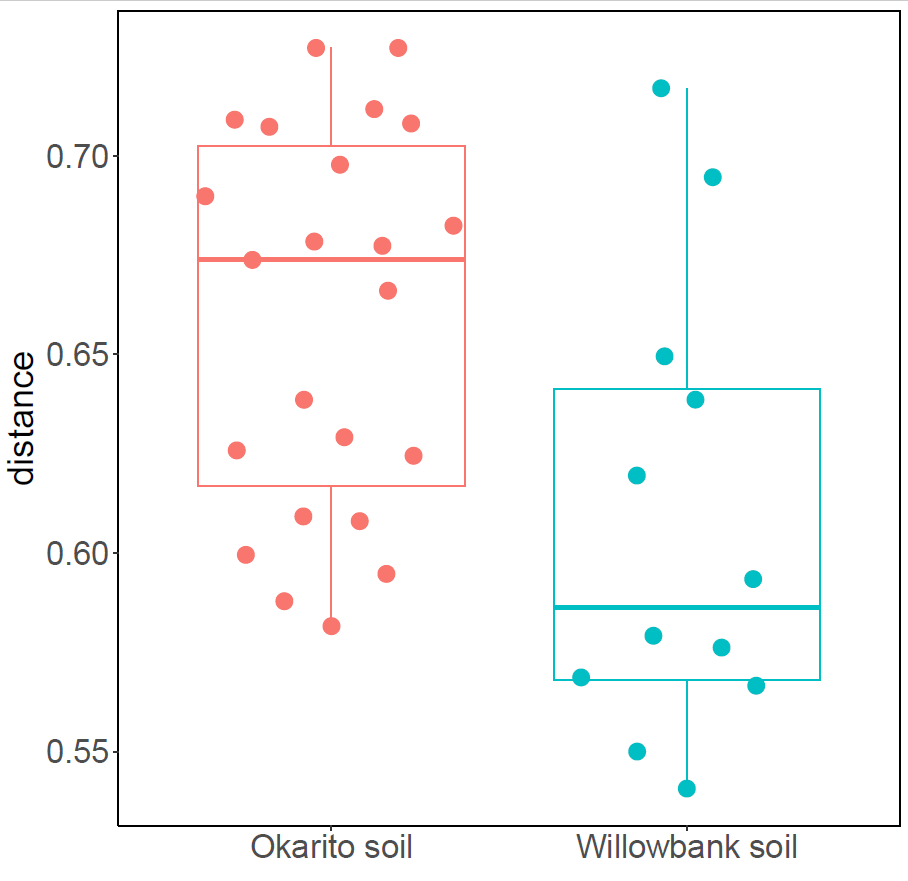


**A)**

**B)**

Distance to Centroid

Distance to Centroid

### **Figure S5** – Fungal beta diversities of Runs stage rowi and major soil types (Ōkārito and WWR). **A)** Mean distance from centroid for both Treatment and Control rowi in the Runs stage was high, indicating both groups were diverse in terms of fungal taxon representation. A minorly significant difference between the two cohorts was found using a Tukey’s multiple comparison of means (p=0.042, TMCM). **B)** Ōkārito soils display a larger distance from centroid across samples compared to WWR soils (p=0.008, TMCM) indicating that individual Ōkārito soil samples were more diverse within-group than WWR soils.


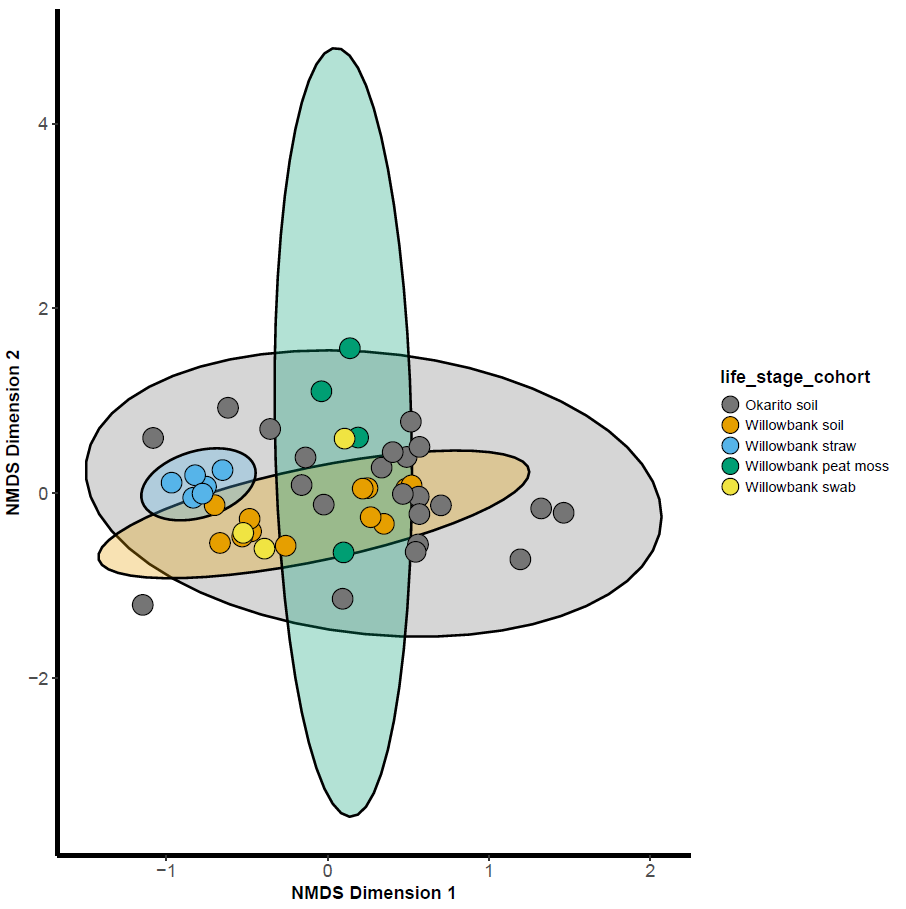


Stress = 0.13


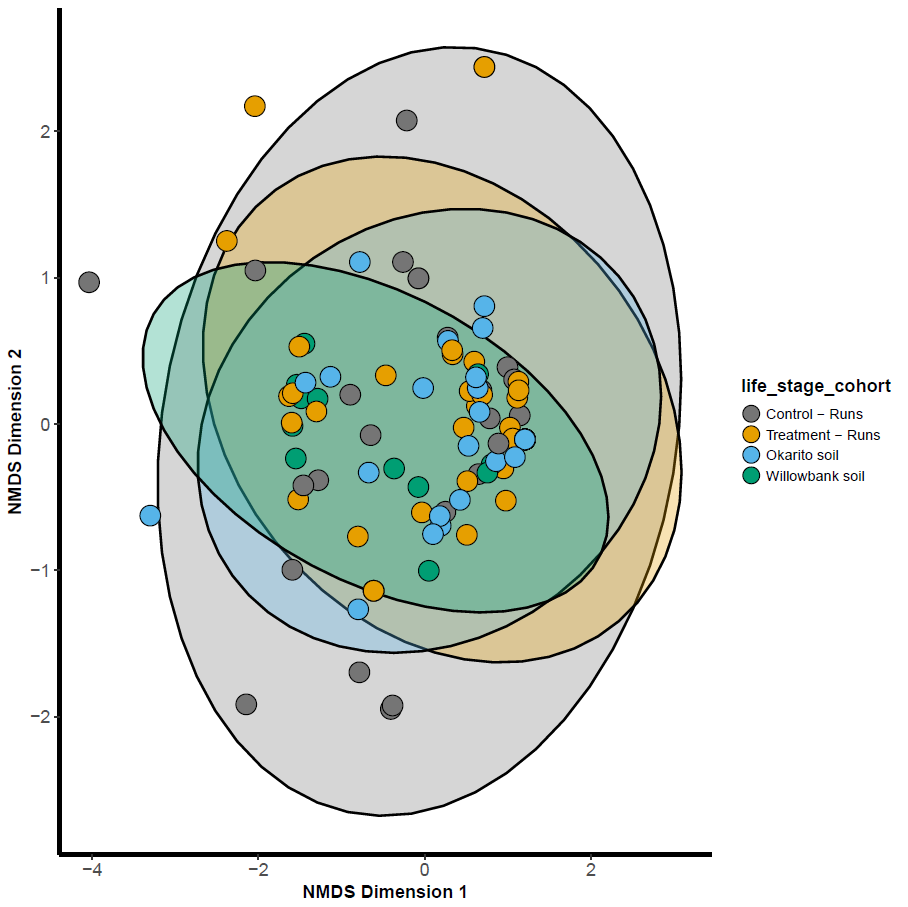


Stress = 0.10

**Cohort and Soil Type**

**Soil Type**

**B)**

**A)**

**Figure S6** – Bray-Curtis dissimilarities of Runs stage rowi organised by cohort, and major soil groups. Ellipses denote grouping at a 95% confidence level. **A)** Major soil groups shared similarities while WWR straw was distinctly clustered with little overlap, indicating a separate fungal microbiome was present in straw. **B)** No clear separation between rowi Control and Treatment birds at the Runs stage was identified. All groups analysed overlapped heavily with one another. Rowi from both cohorts appeared to be more variable in composition than the major soils.


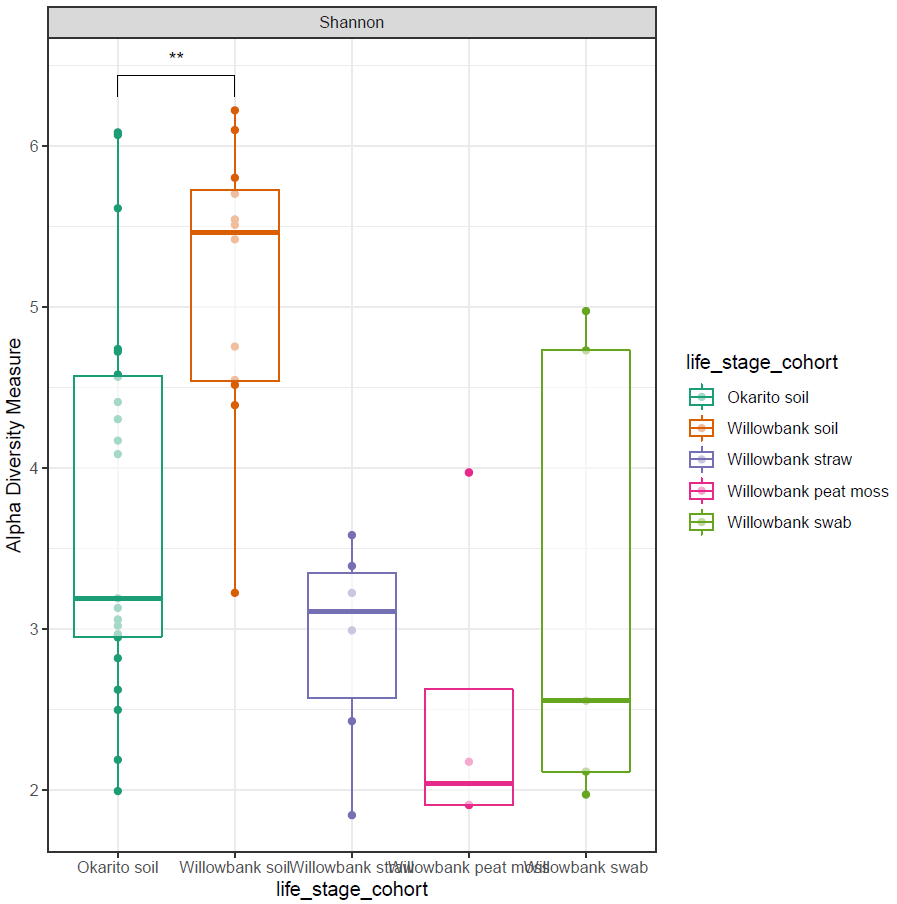


**Soil Type**

Ōkārito soil

WWR soil

WWR straw

WWR peat moss

WWR swabs

Shannon Diversity

**Figure S7**. Shannon alpha diversity of bacterial communities in soil colour-coded by soil group. A) WWR soil displayed high taxonomic diversity within samples when compared with other soils and substrates. A significant difference in alpha diversity was found between Ōkārito soil and WWR soil as denoted by ** (*p* = 0.001, TMCM).

**Figure S8** Shift in bacterial beta diversity variance (distance to centroid) by age in control and treatment cohort birds.

### **
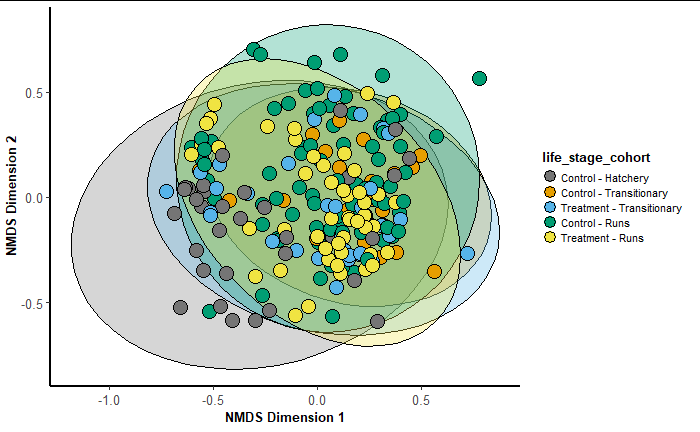
**

**Figure S9** Beta diversity of rowi gut bacterial communities by cohort and life stage (top) and variance of beta diversity (distance to centroid) for rowi gut bacterial communities by cohort and life stage (bottom).

**Supplementary Tables**

### **Table S1** Growth rate statistics *Treatment* vs *Control*


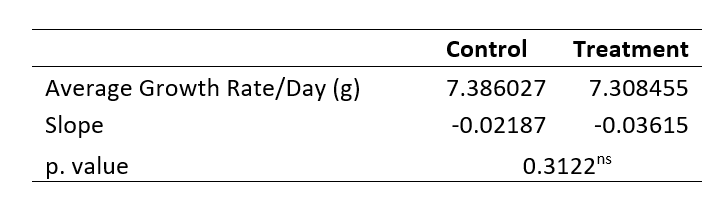


(g/day)
